# Transient hypoglycemia as a rare cause of recurring transient loss of consciousness: a case report 

**DOI:** 10.1186/s13256-021-02844-z

**Published:** 2021-05-06

**Authors:** Michael Wester, Tanja Bergmann, Martina Müller-Schilling, Lars S. Maier, Samuel T. Sossalla

**Affiliations:** 1 Department of Internal Medicine II, University Medical Center Regensburg, Franz-Josef-Strauß-Allee 11, 93053 Regensburg, Germany; 2 Department of Internal Medicine I, University Medical Center Regensburg, Franz-Josef-Strauß-Allee 11, 93053 Regensburg, Germany

**Keywords:** Syncope, Hypoglycemia, Transient loss of consciousness

## Abstract

**Background:**

Syncopes and transient loss of consciousness affect a large number of patients. Determining the underlying mechanism of a syncope is key to effectively treating and preventing future events. However, given the broad differential diagnosis of transient loss of consciousness, it can be challenging to determine the exact etiology.

**Case presentation:**

This case presents a 42-year-old Caucasian female patient with recurrent transient loss of consciousness due to a hitherto undiagnosed impaired glucose tolerance and hyperinsulinism. The patient had been thoroughly tested for all typical causes of syncope without finding any causal explanation. An oral glucose tolerance test confirmed rapidly dropping blood glucose levels associated with rapidly fading consciousness as the underlying cause of transient loss of consciousness. Further diagnostic workup revealed that the patient suffered from impaired glucose tolerance and subsequent hyperinsulinism without overt diabetes mellitus. Nutritional counseling including reduction of glucose intake and frequently eating smaller meal portions led to a significant reduction in the frequency of transient loss of consciousness and overall improvement in quality of life.

**Conclusions:**

The current European Society of Cardiology (ESC) guideline on syncope does not list hypoglycemia as a cause of transient loss of consciousness. However, this case report stresses that metabolic dysregulation can indeed lead to self-limited transient loss of consciousness. Thus, in the case of recurrent syncope with an unclear underlying mechanism, physicians should consider transient hypoglycemia and metabolic workup as a possible differential diagnosis.

## Background

Transient loss of consciousness (TLOC) is a state of real or apparent loss of consciousness with loss of awareness, amnesia for the period of unconsciousness, abnormal motor control, loss of responsiveness, and short duration [1]. TLOC can be grouped into TLOC due to head trauma and nontraumatic TLOC, which includes epileptic seizures, psychogenic causes, and rare causes (for example subclavian steal syndrome, subarachnoid hemorrhage), and syncope [1]. Syncopes are defined as self-limited TLOC due to cerebral hypoperfusion [1]. Syncopes represent a highly relevant disorder due to their high lifetime prevalence and deleterious effect on quality of life [1]. Finding and effectively treating the underlying cause of syncopes or TLOC can be challenging due to the many shared clinical features with other disorders. The current European Society of Cardiology (ESC) guideline on syncope presents a thorough differential diagnosis paired with etiological explanations [1]. However, metabolic disorders are only acknowledged as causing persistent and not self-limiting loss of consciousness. In this case, we present a 42-year-old female patient who suffered from recurrent TLOC labeled as syncopes. Despite extensive workup, no cardiac cause could be determined. In contrast, reactive hyperinsulinism was found to cause real TLOC.

## Case presentation

The 42-year-old Caucasian female patient was hospitalized for further evaluation because frequent TLOC in recent weeks had resulted in drastically reduced quality of life and potentially harmful incidents. TLOC had started appearing 11 months earlier (see “Timeline”), right after she had been hospitalized for a herpes zoster oticus infection with involvement of the vestibular nerve. Ten months earlier, she had experienced three symptomatic hypertensive emergencies with headaches and impaired vision. She had been prescribed 2.5 mg bisoprolol daily; however, compliance was poor, as she reported significant fatigue as a side effect. The patient reported recurrent TLOC especially during light exercise such as walking. In some instances, prodromes such as dizziness and sweating preceded TLOC; other times they occurred suddenly, leading to falls including serious injury. Dyspnea or angina pectoris-like symptoms were not present.

During presentation, we initially took a detailed history and were able to establish that she indeed suffered from TLOC during these episodes. She also reported long-lasting psychosocial distress as she and her partner had been suffering from unintended childlessness. This had fortunately been resolved when they adopted a child 13 months ago.

The physical examination did not show any abnormalities. Height was 174 cm, weight 64 kg, body mass index (BMI) 21.1 kg/m^2^. Heart rate was regular and palpable symmetrically in all extremities (67 beats per minute), and blood pressure was 130/75 mmHg, with no heart murmurs, normal auscultation of the lungs, and no peripheral edema. The abdominal examination revealed no masses and no tenderness.

Recommended basal evaluation of syncopes and TLOC including history, physical examination, electrocardiography (ECG), blood pressure measurements, and orthostatic testing did not yield any pathological findings.

We focused on further evaluating a potential cardiac cause of TLOC. A treadmill cardiac stress test showed normal physical fitness, no arrhythmias, normal blood pressure and heart rate response, and the absence of relevant ST changes. A 24-hour Holter ECG revealed no pathological findings. To account for rare causes of syncopes, Brugada syndrome was ruled out by performing an ajmaline test. Echocardiography showed normal ejection fraction and no valvular dysfunction or other structural disease. No hint of arrhythmogenic right ventricular cardiomyopathy could be detected in cardiac magnetic resonance imaging (MRI). Urinary levels of catecholamines and their metabolites were normal.

**Fig. 1:**
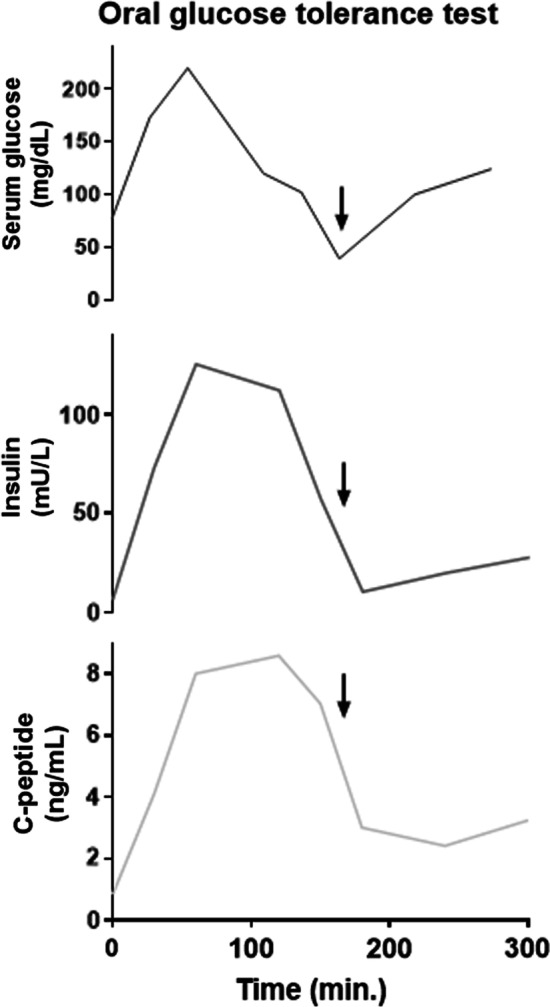
Values of serum glucose, insulin, and C-peptide during the oral glucose tolerance test after ingestion of 75 g glucose. Arrow indicates onset of symptoms of presyncope and administration of intravenous glucose to prevent loss of consciousness.

Upon repeated history-taking about preceding situations and potential triggers, we could elaborate on an association with the intake of carbohydrate-rich foods. The patient reported that the TLOC followed carbohydrate-rich meals by approximately 1–2 hours. She had already intuitively changed her diet by abstaining from carbohydrate-rich meals. To evaluate the association with meals, we performed an oral glucose tolerance test. One hundred and eighty minutes after ingestion of 75 g of glucose, the serum glucose level dropped to 39 mg/dL (see Fig. 1), which induced the same aura symptoms that were familiar to the patient from her previous TLOC with dizziness and rapidly fading consciousness. To prevent complete loss of consciousness, glucose was injected intravenously. Taken together, this provocation test and the detailed medical history proved the hypoglycemic etiology. We therefore established the diagnosis of a pathological glucose tolerance and hyperinsulinism resulting in hypoglycemia and TLOC.

Magnetic resonance imaging (MRI) showed a normal pancreas. A 72-hour-fasting glucose test had been performed and insulinoma had been ruled out. Insulin-like growth factor 1 (IGF-1) levels were normal. Cortisol showed normal levels in serial testing throughout the day. There was no hint of adrenal insufficiency, with normal morning cortisol of 16.2 µg/dL. No insulin antibodies were measurable. Noninsulinoma pancreatogenous hypoglycemia syndrome (NIPHS) is a very rare disease causing postprandial hypoglycemia. Testing includes stimulation of pancreatic β-cells by invasive calcium administration, [2] which was not performed in our patient. Given the relief of symptoms after lifestyle and dietary changes, further invasive testing to distinguish prediabetes from NIPHS was not performed. We recommended primarily dietary changes including frequent small meals to reduce the frequency of hypoglycemic episodes and avoidance of carbohydrate-rich meals as treatment. We discontinued the β-blocker therapy to avoid interference with glycogenolysis. If symptoms persisted, an off-label metformin therapy for the treatment of her pathological glucose resistance or a treatment test with acarbose, which delays hydrolysis of ingested complex carbohydrates and reduces postprandial glucose and insulin peaks, was suggested. At a follow-up consultation 3 months later, the patient reported no further TLOC after the suggested dietary changes, which led to a markedly improved quality of life.

## Discussion

Determining the cause of syncopes and TLOC can be challenging given the broad differential diagnosis. However, identification of the underlying mechanism is crucial to provide optimal treatment and thereby reduce the rate of hospitalization to improve quality of life [3, 4]. Lifetime prevalence of at least one syncope reaches almost 50% [1]. The most common cause of syncopes is reflex syncope, causing about 20% of all syncopes, followed by cardiac and orthostatic syncopes, each accounting for about 10% of all syncopes [3]. However, in more than one third of all patients, no clear etiology can be identified [3, 5]. The current ESC guidelines list metabolic disorders including hypoglycemia as a possible false diagnosis for syncope, stating that metabolic changes produce impairment of consciousness instead of loss thereof and produce a much longer duration than the typically short TLOC in syncope [3]. Contrary to this statement, we hereby report that hypoglycemia can indeed lead to a short, self-limiting loss of consciousness, hence perfectly imitating a syncope.

In this case, the differential diagnosis was complicated as the patient was suffering from various different conditions that may also cause TLOC, dizziness, or vertigo (for a summary of differential diagnosis and rule-out criteria in this patient, see Table 1). These include herpes zoster infection with involvement of the vestibular nerve [6], psychogenic non-epileptic seizures [7], hypertensive emergencies [8], and histamine intolerance [9]. Cardiac syncopes represent a common etiology of TLOC and can be caused by potentially life-threatening pathologies [3]. Intensive electrophysiological and imaging studies to reveal structural diseases could rule out a cardiac cause for the syncopes.

**Table 1:** Differential diagnoses in our patient showing possible symptoms of the different pathologies that can cause or mimic transient loss of consciousness or syncopes

Differential diagnosis	Possible symptoms	Rule-out criteria
Herpes zoster oticus	Vertigo	Patient could clearly differentiate between the vertigo and the syncopes
Hypertensive emergency	Dizziness, impaired vision, headaches	No loss of consciousness during hypertensive episodes
Psychogenic non-epileptic seizure	Unintended childlessness as a stressor	Syncopes occurred after stressor had been relieved
Histamine intolerance	Flush symptoms (heat, sweating, dizziness)	Complete avoidance of histamine-rich food for more than 1 year
Cardiac syncope	Syncope with or without prodromes	No structural heart disease detected in echocardiography or magnetic resonance imaging; normal electrocardiogram and normal treadmill test, no pathological ajmaline test, no arrhythmias in telemetric monitoring

We also included the rule-out criteria for each pathology in our patient

Loss of consciousness due to hypoglycemia occurs almost exclusively in older, long-term diabetic patients, especially in combination with antidiabetic medication such as sulfonylurea or insulin [10–12]. Despite still being under debate, reactive hypoglycemia after ingestion of especially carbohydrate-rich food due to increased peripheral insulin resistance and disturbed insulin secretion has been suggested in prediabetes [13, 14]. In conclusion, given its high prevalence, prediabetes and subsequent dysregulation of insulin secretion causing transient hypoglycemia is the most likely cause of the TLOC in our patient. As TLOC sometimes occurred suddenly without any prior aura, the use of a device for continuous real-time glucose monitoring could be advised to further improve quality of life by preventing harmful accidents.

## Conclusions

In summary, the case of this 42-year-old female patient shows the complexity and difficulties of the workup in patients presenting with recurrent syncope. Hypoglycemic episodes in the absence of manifest diabetes can be a rare differential diagnosis for syncopes and should be considered in patients without conclusive workup. Hypoglycemia usually produces prolonged loss of consciousness. To our knowledge, this is the first case describing transient, self-limiting hypoglycemia as a cause for TLOC. This case stresses the importance of thorough history-taking and metabolic workup in patients presenting with syncopes or TLOC.

## Data Availability

Not applicable.

## Timeline: Organized timeline of the patient’s symptoms and treatments

**Table:**

Time	Events
12 months prior to presentation	A herpes zoster oticus infection caused strong vertigo
11 months prior to presentation	Three singular hypertensive emergencies led to dizziness, vertigo, and impaired vision
11 months prior to presentation	Attacks of transient loss of consciousness (TLOC) started to occur
6 months prior to presentation	Ambulant testing for cardiogenic syncopes including electrocardiography and echocardiography showed normal results
1–2 months prior to presentation	Frequency of TLOC increased to several times per week, causing a drastic impairment of quality of life
Clinical presentation	Rule-out of cardiogenic causes for TLOC; transient hypoglycemia after glucose ingestion due to hyperinsulinism established as cause of TLOC; change of diet suggested
3 months after clinical presentation	No further TLOC after change of diet to 4–5 small meals throughout the day

## Abbreviations

ECG: Electrocardiogram
MRI: Magnetic resonance imaging
NIPHS: Noninsulinoma pancreatogenous hypoglycemia syndrome
TLOC: Transient loss of consciousness
